# Simvastatin Significantly Reduced Alcohol-Induced Cardiac Damage in Adolescent Mice

**DOI:** 10.1007/s12012-023-09821-6

**Published:** 2024-01-23

**Authors:** Makgotso Nchodu, Alice Efuntayo, Robin du Preez, Hasiena Ali, Oladiran I. Olateju

**Affiliations:** School of Anatomical Sciences, Faculty of Health Sciences, University of the Witwatersrand, 7 York Road, Parktown, Johannesburg, 2193 Republic of South Africa

**Keywords:** Alcohol, Adolescents, Simvastatin, Cardiomyocyte morphometry, Fibrosis, Inflammation, Cardiovascular disease

## Abstract

Alcohol abuse by adolescents is becoming a serious health concern as they often progress to becoming alcoholics later in life which may lead to heart problems. Chronic alcohol use alters the cardiac function and structure, such as haemodynamic changes, weakening and loss of cardiomyocytes, myocardial fibrosis, and inflammation. Simvastatin is a commonly used drug for the treatment and management of various cardiovascular problems but information on its protective effects against alcohol-induced cardiomyocyte hypertrophy, fibrosis, and inflammation is lacking in the literature. Four-week-old male (*n* = 5) and female (*n* = 5) C57BL/6 J mice were assigned to each experimental group: (I) NT—no administration of alcohol or Simvastatin; (II) ALC—2.5 g/Kg/day of 20% alcohol via intraperitoneal injection (i.p.); (III) SIM—5 mg/Kg/day of Simvastatin via oral gavage; (iv) ALC + SIM5—5 mg/Kg/day of Simvastatin via oral gavage followed by 2.5 g/Kg/day of 20% alcohol via i.p.; and (v) ALC + SIM15—15 mg/Kg/day Simvastatin via oral gavage followed by 2.5 g/Kg/day of 20% alcohol via i.p. After the 28-day treatment period, the heart was removed and processed for H&E, Masson’s trichrome, or TNF-α immunolabelling. The area and diameter of cardiomyocytes were measured on the H&E-stained sections. The distribution of collagen or TNF-α expression was quantified using the deconvolution tool of ImageJ software. The results confirmed alcohol-induced toxicity on the cardiomyocytes and Simvastatin reduced alcohol-induced cardiomyocyte hypertrophy, fibrosis, and inflammation in both sexes. This study demonstrated that Simvastatin, an FDA approved and easily accessible drug, may be beneficial in lowering the prevalence of alcohol-induced cardiovascular diseases (especially in adolescents) which will have a huge financial implication on health systems worldwide.

**Supplementary Information:**

The online version contains supplementary material available at 10.1007/s12012-023-09821-6.

## Introduction

Adolescence is a period of growth and development characterized by physical, psychosocial, and psychological changes [1, 2]. At this age, adolescents tend to experiment with alcohol resulting in heavy episodic drinking, especially where there are lapses in policies to regulate access to alcohol [1, 2]. Alcohol misuse is higher in male than female adolescents and individuals that begin drinking during adolescence are more likely to become alcoholics later in life [3]. Apart from the social economic impact of alcohol abuse, alcohol misuse causes several metabolic diseases which include cardiac, renal, and hepatic diseases [2–6].

Chronic alcohol use increases the incidence of cardiovascular diseases, which is a huge financial burden on health systems worldwide [4, 7]. Alcohol abuse changes the cardiac function and structure which may result in the weakening and loss of cardiomyocytes [8]. In addition, the mitochondria, plasma membrane, ribosomes, and receptors of cardiomyocytes are also damaged due to the high reactivity, high diffusion rate, and the ability of alcohol to easily cross membranes [9]. Alcohol furthermore disrupts signalling processes, activates apoptosis, and decreases heart contraction [7, 9]. Likewise, the repair mechanism of cardiomyocytes is hindered by alcohol (i.e. by reducing regeneration and proliferation of cardiomyocytes) whilst promoting cardiac lesions (i.e. by increasing apoptosis and necrosis of cardiomyocytes), the outcome of which leads to myocardial fibrosis [9] and ventricular dysfunction [10]. Alcohol is also known to induce inflammation and promote the secretion of cytokines such as tumour necrosis factor-α (TNF-α) [7], a pro-inflammatory cytokine that regulates myocardial homeostasis by signalling inflammation and cell abnormality (e.g. apoptosis) [7]. It must be noted that an increased level of TNF-α in chronic alcoholism is an indication of cardiac pathologies, fibrosis, and necrosis [7, 11, 12].

With the prevalence of chronic alcoholism (especially in adolescents) and its associated cardiovascular diseases, it is important to investigate interventions that may mask or reduce the damaging effects of alcohol on the heart tissues. One such intervention that is gaining popularity, due to its anti-inflammatory and immune-regulatory properties, is Simvastatin [13–15], an FDA-approved drug, primarily used for lowering blood cholesterol levels (hypercholesterolemia) [16–19]. Simvastatin has also been shown to prevent coronary heart disease [19, 20], cardiomyocyte death [21], myocardial inflammation, and fibrosis [14, 22–24]. Furthermore, Simvastatin also prevents myocarditis [25], improves endothelial functions [14, 15], and inhibits the TGF-β1 pathway which triggers the development of interstitial fibrosis and cardiomyocyte hypertrophy [14].

Simvastatin may therefore be beneficial against alcohol-induced myocardial damage by preventing or potentially reversing the structural alterations caused by chronic alcohol use. Thus, this study investigated the protective capabilities of Simvastatin against alcohol-induced damage on the heart of adolescent mice that were administered alcohol. Preventing and/or reversing alcohol-induced damage could alleviate the financial burden caused by alcohol-related diseases [4].

## Materials and Methods

### Experimental Animal Study

This study received ethical approval (Ethics Clearance No: 2019/11/63/C) from the Animal Research Ethics Committee (AREC) of the University of the Witwatersrand Johannesburg, South Africa. Mice of the same sex and belonging to the same experimental group were housed together in a group of five mice per cage (cage dimensions: 200 × 200 × 300 mm) and kept under a reversed 12–h-day/12-h dark cycle (with the light switched off from 06:00 to 18:00). For this study, the period of adolescence in mice was taken as the period between 4 and 8 weeks old [26].

Four-week-old male (*n* = 25; 5 per group) and female (*n* = 25; 5 per group) C57BL/6 J mice housed in the Witwatersrand Research Animal Facility (WRAF), University of the Witwatersrand were assigned to each experimental group: (I) Non-treatment group (NT); (II) Alcohol-only group (ALC)—2.5 g/Kg/day of 20% alcohol via intraperitoneal injection (i.p.); (III) Simvastatin-only group (SIM)—5 mg/Kg/day Simvastatin via oral gavage; (IV) Alcohol + Simvastatin 5 (ALC + SIM5)—5 mg/Kg/day of Simvastatin via oral gavage followed by 2.5 g/Kg/day of 20% alcohol via i.p.; and (V) Alcohol + Simvastatin 15 (ALC + SIM15)—15-mg/Kg/day Simvastatin via oral gavage followed by 2.5 g/Kg/day of 20% alcohol via i.p. Simvastatin (Cat no: 1612700, Merck, South Africa) was prepared according to McKay et al. [27] to ensure complete dissolution. In addition, a pharmacological-grade absolute alcohol (99.9%) (Sigma-Aldrich, South Africa; Cat No: 24105) was serially diluted in saline (0.9% NaCl) into a 20% alcohol solution. Both Simvastatin and alcohol were prepared daily and then filter sterilized before administering. Any unused solution per day was discarded. All treatments were performed for 28 consecutive days. Oral gavage and i.p. were performed with the utmost care by the trained staff of WRAF to reduce introducing stress into the animal.

On the last day of treatment, 50-μL saphenous blood was collected in heparinized capillary tubes within 30 min after the administration of alcohol before being centrifuged with Vivaspin500© 100-μm membrane tubes (Biotech, South Africa) at 5000 rpm for 15 min to separate the serum. The serum was extracted, and the Blood Alcohol Concentration (BAC) was determined using an EnzyChrom™ Ethanol Assay Kit (BioVision, South Africa). The average BAC level was in the range of 182.5–253.4 mg/dL for the groups that were administered alcohol.

Following blood collection, the mice were euthanized using Euthanaze (sodium pentobarbital, 80 mg/Kg, i.p.). Thereafter, the mice were transcardially perfused with 4% paraformaldehyde (in 0.1-M phosphate buffer, PB) (PFA), the heart was removed, and then further fixed in 4% PFA at 4 °C before further processing.

### Tissue Processing and Morphometries

The PFA-fixed heart was cut midway across the ventricles. The lower portion of the ventricles was processed for histology and sectioned horizontally across the ventricles at 2 µm thickness. Serial sections were prepared for haematoxylin and eosin (H&E) staining (for general morphology and morphometry of cardiomyocytes), Masson’s trichrome (MT) staining (for evaluating myocardial fibrosis), or TNF-α immunolabelling (for quantifying the expression of TNF-α; primary antibody—1:250, mouse anti-TNF-α, ab220210, Abcam; secondary antibody—1:1000, goat anti-mouse IgG, BA-9200–1.5, Vector labs).

### Cardiomyocyte Morphometry

For the morphometries of the cardiomyocytes, images of the myocardium along the length of the left ventricular wall of the H&E-stained sections were taken using an Olympus EP50 camera (Serial No. 3H23424, Japan) attached to an Olympus BX41 microscope (Model BX41TF, Olympus Corporation, Tokyo, Japan) at times 100 objective lens (under oil immersion). The left ventricular wall was identifiable as the larger of the two ventricular walls. The inter-ventricular wall was not considered in this study. With the scale set on the software, the area and the diameter of the cardiomyocytes [28] were measured from the digitized image using EPview software (EPview 1.3, Build 19,857). The diameter of the long axis of a cardiomyocyte was measured across the level of the nucleus with the line passing through the nucleolus using the straight-line tool of the software [28, 29]. In addition, the border of cardiomyocyte was carefully traced using the polygonal tool of the software and whereafter the cardiomyocyte area was analysed [28, 29]. The field of view was changed by moving the microscope stage at regular intervals to prevent duplicating measurements.

### Quantification of Collagen Distribution

To determine the distribution of collagen deposition in the myocardium, digitized images of the left ventricular wall of the MT-stained sections were taken using a Carl Zeiss Axiocam 208 colour camera (Serial No. 5318003446, China) attached to a Carl Zeiss Axioskop 2 microscope (Serial No. 804161, Germany) at times 40 objective lens. Similar to the cardiomyocyte morphometry, the field of view was changed by moving the microscope stage along the length of the left ventricular wall. The distribution of collagen within the myocardium was determined using the deconvolution plug-in settings on ImageJ software (ImageJ 1.53q/Java 1.8.0_322, Oracle America Inc.). The 24-bit RGB format was selected as a requirement for the deconvolution plug-in setting in the ImageJ software where the green component on the processed image indicated collagen stain. Collagen distribution on each image was quantified using the ImageJ software’s threshold tool, which was adjusted until all the collagen (i.e. green) stains had been highlighted [30, 31]. The percentage collagen distribution per image was calculated as the threshold area divided by the area of the image.

### Quantification of TNF-α Distribution

Like the analyses used for the MT stain sections, the percentage distribution of TNF-α-positive immunolabelling in the myocardium was performed using digitized images at 40 times objective lens. The field of view was also changed before processing with ImageJ software. Using the 24-bit RGB format, 3,3’Diaminobenzidine (DAB) was selected for the deconvolution plug-in setting on the ImageJ software where the brown component was identified as DAB staining. The distribution of TNF-α immunolabelling in each image was quantified by adjusting the threshold tool of ImageJ until all the DAB stains had been highlighted [32]. Thereafter, the percentage distribution of TNF-α immunolabelling was determined by dividing the threshold area by the area of the image.

### Statistical Analyses

A descriptive statistic using the mean (± SD) and the median was performed. Normality test was conducted using the Shapiro–Wilk test and then either One-Way ANOVA or Kruskal–Wallis test was conducted to compare the mean or median of the measurements across the different groups. A Post hoc test using either a Tukey’s or a Dunn’s test was conducted to determine where significant difference lies between any two groups. All statistical tests were performed using a PAST freeware data analyser (version 4.03; Germany) and graphs were plotted using Excel software (Word Office Pro, USA). A statistical difference of 5% was regarded as significant for all statistical analyses.

## Results

### General Observation and Morphology

No observable physical abnormalities (e.g. a reduced body mass) relating to the adverse effects of alcohol were detected in any of the mice. At the time of sacrifice, the average body mass, average heart mass, and the heart/body mass ratio of the mice are shown in Table 1. There was a significant difference in the heart/body mass ratio across the groups in the females but there was no difference in the males.

**Table 1:** A summary of the body mass, the heart mass, and the heart/body mass ratio of mice across the different groups

	NT	SIM	ALC	ALC + SIM5	ALC + SIM15
Female					
Body mass	14.5 ± 1.58	14.80 ± 1.15	14.6 ± 1.24	13.9 ± 1.02	15.3 ± 1.89
Heart mass	0.26 ± 0.03	0.18 ± 0.09	0.28 ± 0.04	0.15 ± 0.05	0.27 ± 0.07
Organ/body ratio	0.018 ± 0.001^a^	0.012 ± 0.007^b^	0.019 ± 0.003^b,c^	0.011 ± 0.003^a,c,d^	0.017 ± 0.002^d^
Male					
Body mass	18.9 ± 1.67	18.4 ± 1.64	18.03 ± 1.66	18.1 ± 0.55	15.9 ± 1.39
Heart mass	0.24 ± 0.11	0.25 ± 0.09	0.31 ± 0.04	0.34 ± 0.03	0.25 ± 0.14
Organ/body ratio	0.013 ± 0.006	0.013 ± 0.005	0.017 ± 0.002	0.019 ± 0.001	0.016 ± 0.008

*SD* standard deviation

^a,b,c,d^The same letter indicates paired groups that are statistically significantly different at *P* < 0.05

**Fig. 1:**
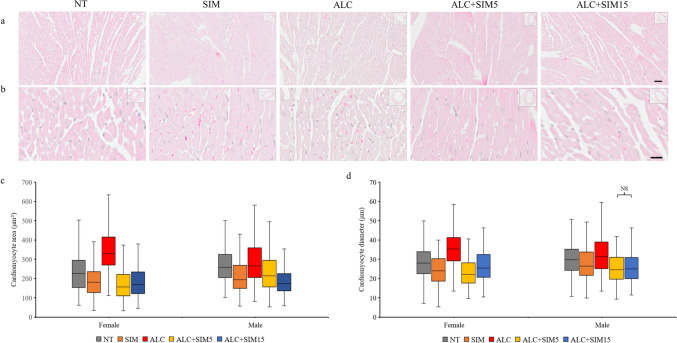
Representative photomicrographs of the H&E-stained sections **a** at a low magnification (Scale bar = 50 µm), **b** at a high magnification (Scale bar = 20 µm), the box plots of **c** the cardiomyocyte area, and **d** the cardiomyocyte diameter across the different experimental groups, for both sexes. The histology of the myocardium was typical of the normal histology found in the myocardium of mice. The cardiomyocytes were oval with distinct nuclei. In both sexes, the area or the diameter of cardiomyocytes was significantly higher in the ALC group than in the NT group, thus confirming alcohol-induced myocardial hypertrophy. Both concentrations of Simvastatin were effective against alcohol-induced myocardial hypertrophy in both sexes. No editing was done to the images. *NT* non-treatment, *SIM* 5-mg Simvastatin, *ALC* alcohol, *ALC + SIM5* 5-mg Simvastatin and alcohol, *ALC + SIM15* 15-mg Simvastatin and alcohol, *NS* not significant at *P* > 0.05

**Fig. 2:**
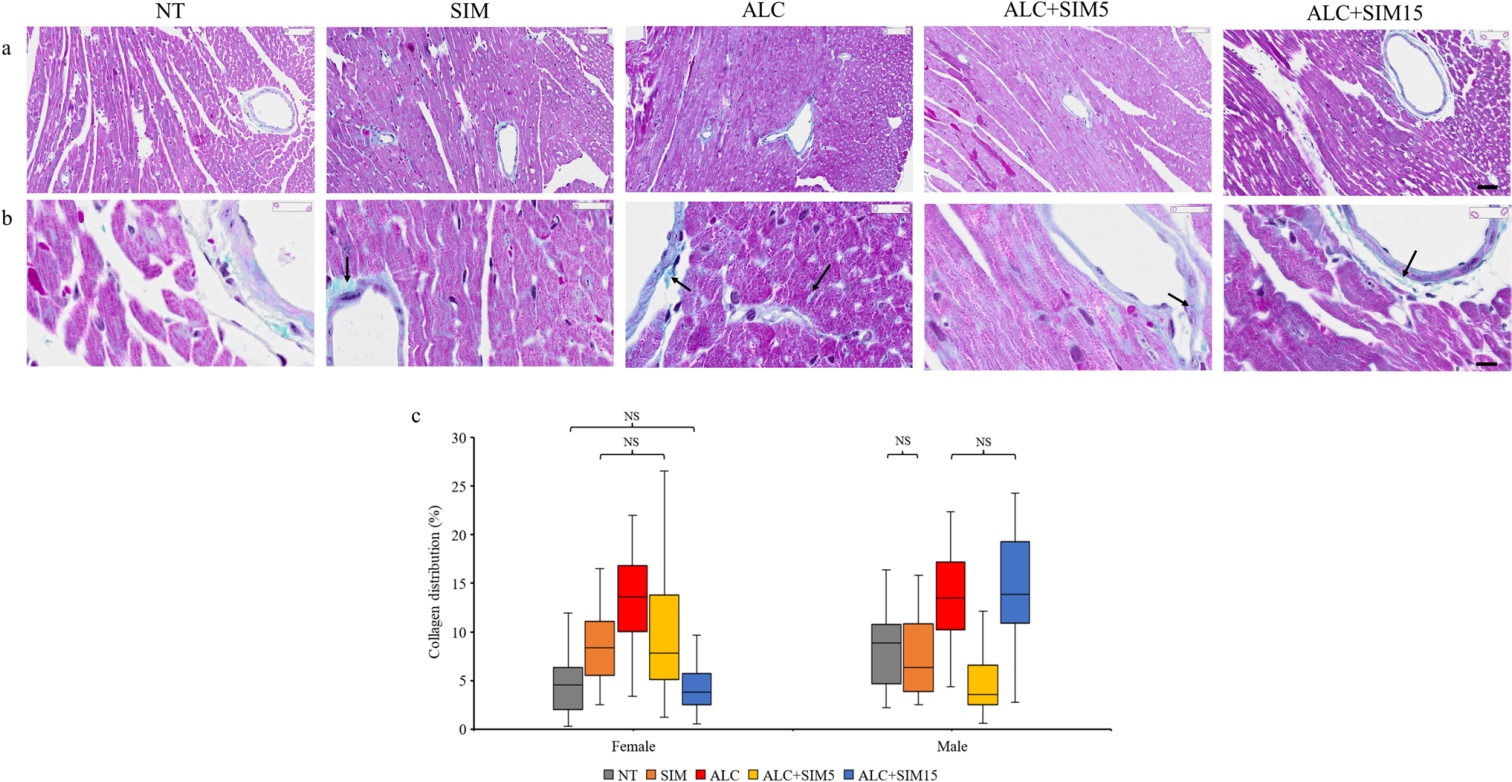
Representative photomicrographs of the MT-stained sections **a** at a low magnification (Scale bar = 50 µm), **b** at a high magnification (Scale bar = 10 µm), and **c** the box plot of the percentage collagen distribution across the different experimental groups, for both sexes. Collagen deposition in the myocardium is indicated by black arrows. In both sexes, collagen distribution was significantly higher in the ALC group than in the NT group, thus confirming alcohol-induced myocardial fibrosis. Both Simvastatin concentrations significantly reduced alcohol-induced myocardial fibrosis except in the males where 15-mg Simvastatin had no beneficial effect—an indication of possible sex-specific differences in the effects of Simvastatin against alcohol-induced myocardial fibrosis. No editing was done to the images. *NT* non-treatment, *SIM* 5-mg Simvastatin, *ALC* alcohol, *ALC + SIM5* 5-mg Simvastatin and alcohol, *ALC + SIM15* 15-mg Simvastatin and alcohol, *NS* not significant at *P* > 0.05

**Fig. 3:**
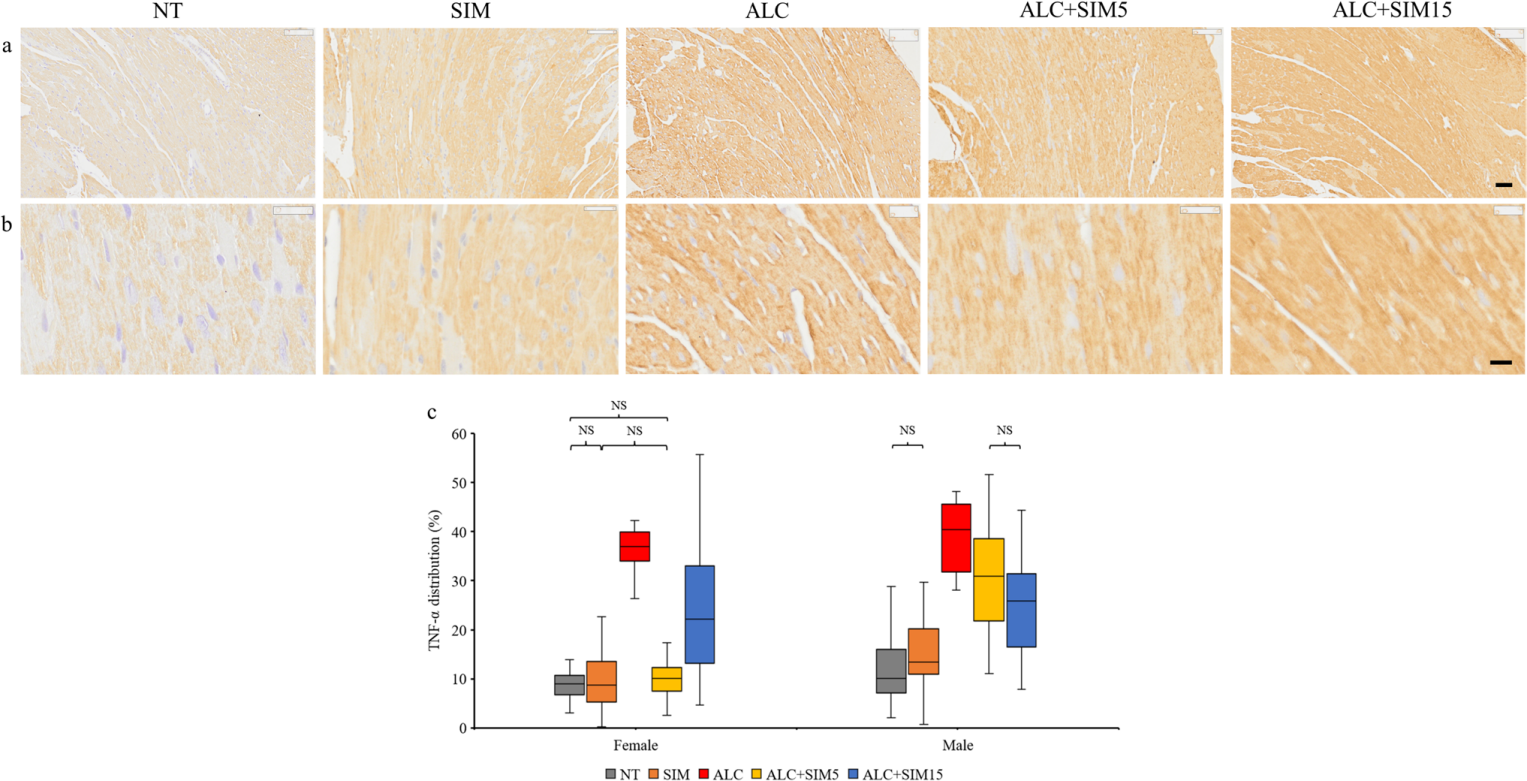
Representative photomicrographs of the TNF-α-immunolabelled sections **a** at a low magnification (Scale bar = 50 µm), **b** at a high magnification (Scale bar = 50 µm), and **c** the box plot of the percentage TNF-α distribution across the different experimental groups, for both sexes. Varying degrees of TNF-α expression are noticeable across the experimental groups. In both sexes, TNF-α distribution was significantly higher in the ALC group than in the NT group, thus confirming alcohol toxicity in the cardiomyocytes. Both concentrations of Simvastatin were effective against alcohol-induced myocardial inflammation. A lower Simvastatin concentration was effective in the females but not in the males—an indication of possible sex-specific differences in the effect of Simvastatin against alcohol effects on the cardiomyocytes. No editing was done to the images. *NT* non-treatment, *SIM* 5-mg Simvastatin, *ALC* alcohol, *ALC + SIM5* 5-mg Simvastatin and alcohol, *ALC + SIM15* 15-mg Simvastatin and alcohol, *NS* not significant at *P* > 0.05

The heart ultrastructure across the experimental groups was typical of normal heart tissue histology and the tissue ultrastructure was similar in both sexes. The cardiomyocytes (in the myocardium) were mostly oval and their nuclei were quite distinguishable as seen in the H&E–stained sections (Fig. 1a and b). A denser collagen staining (indicated by green staining) was observed mostly in the alcohol-only group as shown in the MT-stained sections (Fig. 2a and b). Likewise, a denser TNF-α expression/immunolabelling was more obvious in the alcohol-only group, which indicated inflammation as observed in the TNF-α-immunolabelled sections (Fig. 3a and b).

### Measurements of the Cardiomyocyte Area and Diameter

Descriptive statistics for the cardiomyocyte diameter and area across the different experimental groups of both sexes are shown in Table 2. In the females, both the diameter and the area of cardiomyocytes were highest in the ALC group and lowest in the ALC + SIM5 group. A Kruskal–Wallis test revealed that the cardiomyocyte area or diameter was significantly different across the experimental groups (*P* < 0.000), whilst a Dunn’s post hoc revealed that the cardiomyocyte area or diameter in any paired groups was significantly different (*P* < 0.000) (Fig. 1c and d). The significant increase in cardiomyocyte area or diameter in the ALC group compared to the NT group validates the damaging effect of chronic alcohol use on the cardiomyocytes. Alcohol-induced cardiomyocyte hypertrophy was significantly reduced by both concentrations of Simvastatin; however, the lower concentration (5 mg) seems to be more effective.

**Table 2:** Summary of the morphometries of the cardiomyocyte area and the diameter and the distributions of collagen and TNF-α

		Cardiomyocyte area			Cardiomyocyte diameter			Collagen distribution			TNF-α distribution		
	No of animals	No of cardiomyocytes assessed	Mean ± SD (µm^2^)	Median (µm^2^)	No. of cardiomyocytes assessed	Mean ± SD (µm)	Median (µm)	No of images assessed	Mean ± SD (%)	Median (%)	No of images assessed	Mean ± SD (%)	Median (%)
Female													
NT	5	500	229.94 ± 95.27	226.31	500	28.66 ± 8.02	28.00	43	4.66 ± 2.95	4.57	20	8.74 ± 2.63	8.91
SIM	5	500	189.28 ± 79.37	180.17	500	24.47 ± 7.11	23.95	45	8.75 ± 3.73	8.40	39	9.94 ± 6.25	8.69
ALC	5	500	346.21 ± 105.54	329.83	500	35.51 ± 8.44	35.36	39	13.37 ± 4.77	13.62	40	36.40 ± 4.35	37.03
ALC + SIM5	5	500	170.91 ± 76.23	156.28	500	23.16 ± 7.07	22.11	43	9.70 ± 7.04	7.83	41	9.83 ± 3.84	10.02
ALC + SIM15	5	500	179.15 ± 72.10	168.46	500	26.37 ± 7.38	25.50	41	4.32 ± 2.67	3.81	41	24.50 ± 13.93	22.17
Male													
NT	5	500	270.75 ± 85.52	259.13	500	30.06 ± 7.85	29.73	43	8.25 ± 3.66	8.84	37	11.58 ± 6.54	10.10
SIM	5	500	210.69 ± 78.77	193.97	500	28.00 ± 8.44	26.50	37	7.32 ± 4.04	6.32	26	14.78 ± 8.03	13.67
ALC	5	500	289.32 ± 104.75	265.80	500	32.95 ± 9.96	31.41	38	13.96 ± 4.69	13.52	35	39.13 ± 6.55	40.46
ALC + SIM5	5	500	223.74 ± 85.25	213.88	500	25.27 ± 7.06	24.50	39	4.78 ± 3.03	3.57	34	31.28 ± 11.08	30.87
ALC + SIM15	5	500	184.02 ± 65.11	173.10	500	25.55 ± 6.89	24.94	39	13.70 ± 5.93	13.84	36	24.72 ± 9.71	25.86

*SD* standard deviation

In the males, both the diameter and the area of cardiomyocytes were also highest in the ALC group but lowest in the ALC + SIM15 for the cardiomyocyte area and in the ALC + SIM5 for the cardiomyocyte diameter (Table 2). A Kruskal–Wallis test revealed that the cardiomyocyte area or diameter was significantly different across the experimental groups (*P* < 0.000) and a Dunn’s test also revealed that the area or diameter of cardiomyocytes in any paired group was significantly different (*P* < 0.000) (Fig. 1c and d). The significant increase in cardiomyocyte area or diameter in the ALC group compared to the NT group also validates the damaging effect of chronic alcohol on the cardiomyocytes of adolescent mice. Alcohol-induced cardiomyocyte hypertrophy was significantly reduced by both concentrations of Simvastatin.

### Measurement of Collagen Distribution

In the females, collagen distribution (%) was highest in the ALC group and lowest in the ALC + SIM15 group (Table 2). A Kruskal–Wallis test revealed that the collagen distribution was significantly different across the experimental groups (*P* = 0.000), whilst a Dunn’s test revealed that collagen distribution was significantly higher in the ALC group compared to the NT (*P* = 0.000), SIM (*P* = 0.002), ALC + SIM5 (*P* = 0.001), or ALC + SIM15 (*P* = 0.000) groups. However, collagen distribution was similar in NT vs ALC + SIM15 (*P* = 0.639) groups or SIM vs ALC + SIM5 (*P* = 0.799) groups (Fig. 2c). Similar to the cardiomyocyte hypertrophy, the significant reduction in collagen distribution in the NT group, more than in the ALC group, also validates alcohol-induced myocardial fibrosis. Both concentrations of Simvastatin were also effective against alcohol-induced myocardial fibrosis but the higher concentration (15 mg) seems to be more effective.

In the males, the collagen distribution (%) was also highest in the ALC group but lowest in the ALC + SIM5 group (Table 2). A Kruskal–Wallis test revealed that the collagen distribution was significantly different across the experimental groups (*P* = 0.000) and a Dunn’s post hoc revealed that the collagen distribution was also significantly higher in the ALC group than in the NT (*P* = 0.000), SIM (*P* = 0.000) or ALC + SIM5 (*P* = 0.000) groups. However, collagen distribution was similar in NT vs SIM (*P* = 0.409) groups or ALC vs ALC + SIM15 (*P* = 0.708) groups (Fig. 2c). The significant reduction in collagen distribution in the NT group, more than in the ALC group also validates alcohol-induced myocardial fibrosis. Surprisingly, only the lower concentration of Simvastatin (5 mg) was effective against alcohol-induced myocardial fibrosis, but 15-mg Simvastatin had no beneficial effect against alcohol-induced damage in the males unlike in the females. This is an indication of possible sex-specific differences in the effects of Simvastatin against alcohol-induced fibrosis.

### Measurement of TNF-α Distribution

TNF-α distribution was highest in the ALC group in both sexes (Table 2). A Kruskal–Wallis test revealed that the TNF-α distribution in the females was significantly different across the experimental groups (*P* = 0.000), whilst a Dunn’s post hoc revealed that the TNF-α distribution in any paired groups was significantly different except for the following groups; NT vs SIM (*P* = 0.751), SIM vs ALC + SIM5 (*P* = 0.718), or NT vs ALC + SIM5 (*P* = 0.538) (Fig. 3c). The significant reduction in TNF-α distribution in the NT group than in the ALC group also validates an alcohol-induced myocardial inflammation. Both concentrations of Simvastatin were effective against alcohol-induced myocardial inflammation, but the lower Simvastatin concentration (5 mg) was more effective than the higher Simvastatin (15 mg) concentration.

In the males, a Kruskal–Wallis test revealed that the TNF-α distribution was significantly different across the experimental groups (*P* = 0.000), whilst a Dunn’s post hoc revealed that the TNF-α distribution in any paired groups was significantly different except in the NT vs SIM (*P* = 0.267) groups (Fig. 3c). The significant reduction in TNF-α distribution in the NT group than in the ALC group also validates an alcohol-induced myocardial inflammation. Likewise, both concentrations of Simvastatin were equally effective against alcohol-induced myocardial inflammation; however, the higher concentration was more effective in the males unlike in the females. This is an indication of possible sex-specific differences in the effects of Simvastatin against alcohol-induced inflammation.

## Discussion

Chronic alcohol consumption causes alcoholic cardiomyopathy (i.e. the disruption of the myofibrillary architecture which supports cardiac cells) [33] as well as cardiomyocyte hypertrophy, fibrosis, and inflammation [10, 33–35]. Chronic alcohol use also worsens cardiac function and weakens and promotes the loss of cardiomyocytes [8]. Cardiomyocyte organelles such as the mitochondria, ribosomes, plasma membrane, and membrane receptors are also damaged due to the high reactivity, low molecular mass, high diffusion rate, and high permeability of alcohol [9]. Alcohol permeability is further implicated in the disruption of signalling processes, activation of apoptosis, and the loss of heart contraction [7, 9]. Even acute alcohol exposure has been demonstrated to elicit haemodynamic changes (cardiac stress) leading to the enlargement of cardiomyocytes [7, 33, 36]. However, it must be noted that myocardial hypertrophy (which causes the thickening of the ventricular wall) may occur at the early stages of alcohol damage as a compensatory mechanism for the sarcoplasmic reticular dysfunction (arising from the effect of alcohol) [8, 9, 35, 37]. In a situation where the compensatory mechanism fails, ventricular failure is eminent [35] and causes an increase in circulatory cytokines and growth factors [7].

Likewise, alcohol hinders the repair mechanism of cardiomyocytes by suppressing regeneration whilst promoting cardiac lesions through its ability to trigger apoptosis and necrosis of cardiomyocytes [9]. A hindered repair mechanism promotes myocardial fibrosis [9] disrupting myocardial remodelling which is an essential contributor to the repair of ventricular wall damage [10, 38]. The failure of remodelling after ventricular wall damage leads to ventricular dysfunction [10] characterized by a dilated left ventricle, a thin left ventricular wall, a disrupted myofibrillary architecture, and contractile dysfunction [33, 35, 36]. Ventricular dysfunction in alcoholics is also caused by the disruption of calcium ion homeostasis and its regulation by inhibiting endothelial nitric oxide synthase (eNOS) [38–40].

Alcohol induces inflammation and promotes the secretion of cytokines, such as TNF-α [7]. The upregulation of TNF-α signals inflammation and cell abnormality (e.g. apoptosis), thus it plays a key role in myocardial homeostasis [7]. TNF-α is mainly released by immune cells (e.g. macrophages and T-lymphocytes); however, it is also secreted by cardiomyocytes [41]. This is also the reason why the heart is also called a TNF-α-producing organ, especially during heart failure when there is a surge in secretion [7, 42]. Low levels of TNF-α were reported to prevent minor cardiac injuries but inflammation is exacerbated at high concentrations [41]. Thus, TNF-α regulates autocrine and paracrine activities of the endothelial cells at low concentrations which makes it a crucial monitor of inflammation [41]. Concerning chronic alcohol use, an elevated expression of TNF-α indicates alcohol-related cardiomyocyte damage as is the case in the study presented [7, 11, 12]. It is therefore not surprising that a 28-day chronic alcohol treatment cycle induced myocardial hypertrophy, fibrosis, and inflammation, which confirms the damaging effects of alcohol on adolescent cardiac tissue in the present study.

Generally, Simvastatin is a commonly used drug for treating different cardiovascular diseases, especially hypercholesterolemia [16–19], coronary heart disease [19, 20], cardiac cell death [21], myocardial inflammation, and fibrosis [14, 15, 22, 24, 25, 43] due to the inter-relationships between these diseases. Simvastatin can also inhibit the TGF-β1 pathway which is responsible for interstitial fibrosis and cardiomyocyte hypertrophy [14]. In addition, it has been shown that Simvastatin can prevent cardiac dysfunction and remodelling through its unique ability to stimulate some important pathways, such as Krüppel-like Factor 2 (Klf2) in endothelial cells. It directly regulates the activities of the TGF-β1 target genes in these cells, and this provides non-cholesterol-related evidence of the effects of Simvastatin against cardiac failures [44]. Simvastatin also reduces cardiac hypertrophy due to its ability to upregulate peroxisome proliferator-activated receptors [45] and inhibits autophagy [46]. Simvastatin therefore can protect and prevent damage to the cardiomyocytes [14, 44–49].

In addition, Simvastatin is also effective against several toxins such as cyclophosphamide-induced toxicity via the modulation of inflammasome/caspase1/interleukin 1β [50], lipopolysaccharide-induced myocardial injury by up-regulating survivin that inhibits caspase-3 activation [51] and Ischemia–reperfusion-induced myocardial damage by reducing oxidative stress [52]. Considering the ability of Simvastatin to protect cardiac tissues from being damaged by toxins, it became necessary to explore its effectiveness against alcohol-induced cardiomyocyte damage and it is not surprising that the present study found that both Simvastatin concentrations (5 mg or 15 mg) significantly reduced alcohol-induced cardiomyocyte hypertrophy, cardiac fibrosis, and inflammation, whilst the administration of Simvastatin alone did not seem to be toxic to the cardiac tissues. Unfortunately, there is no report to directly compare the findings of this study, but several studies have shown that higher concentrations of Simvastatin are also beneficial (e.g. Qin et al. [45] at 25 mg/kg/day and Xiao et al. [14] at a range of 10–40 mg/kg/day).

Although the mechanism of action of Simvastatin activity was not explored in the present study, it is assumed that Simvastatin’s ability to modulate several well-researched intracellular activities may have come to play in the process of preventing alcohol damage in the cardiac tissue. It is well documented that Simvastatin actively inhibits the production of mevalonate (MVA), which is responsible for the growth of mammalian cells and the production of cholesterol. MVA is synthesized by the hydroxy-3-methylglutaryl coenzyme A (HMG-CoA) reductase and the metabolism of MVA generates other products with diverse physiological and cellular functions, such as Coenzyme Q in the mitochondrial respiratory chain, farnesyl and geranylgeranyl moieties for protein post-translational modifications, isopentenyl tRNAs for RNA transcription, and dolichol for protein N-glycosylation [53–55]. It has been demonstrated that the ability of Simvastatin to inhibit the MVA pathway is responsible for the anti-hypertrophic, anti-fibrotic, and anti-inflammatory capabilities of Simvastatin [22, 56–69].

In conclusion, this study showed the degree of alcohol-induced cardiac damage and demonstrated the effects of Simvastatin against alcohol-induced cardiac damage in adolescent mice. This novel study is crucial to treating and managing alcohol-related cardiovascular diseases. The present study also highlights possible sex-specific differences in the effects of the different concentrations of Simvastatin against alcohol-induced cardiac damage. This is an important observation that underpins the possible influence of underlying biological factors on the effect of Simvastatin against chronic alcohol use and further studies are required to elucidate these factors. In addition, the use of an FDA approved and easily accessible drug such as Simvastatin may help lower the prevalence of alcohol-related cardiovascular diseases and invariably help reduce the impact of the disease on the economy and health systems worldwide.

### Supplementary Information

Below is the link to the electronic supplementary material.

Supplementary file1 (XLSX 109 KB)

## Data Availability

The raw data for this study is uploaded as a supplementary file.
